# News Items

**Published:** 1869-07-15

**Authors:** 


### NEWS ITEMS.

R. M. Lackey, M.D., formerly of this city, is now editing *The Medical Adviser*, a double column magazine of four pages, 5 in. by 9 in. (including margin) each. The initial number only has reached us. It appears to be published gratuitously. Those wishing copies can address the Editor, P. O. box 35, Lyons, Iowa. Without explanations in subsequent numbers, we should conclude that a quite intelligent gentleman had subsided into the depths of the most pitiable quackery.

Washington City and Louisville are each to have a new Medical Journal, and the latter city is to rejoice in the luxury of a third Medical College, which we suppose will attempt to develop into the Grand National College suggested at the late meeting of the American Medical Asscia-tion. At least a score of schools located in country towns and ambitious little cities are putting in their claims for this distinction, and our Louisville friends must "look alive" if they would secure the honor. The new college has our vote in advance. Small towns are better for philosophical retirement and scholastic pursuits, not to speak of the removal of students from those unspeakable temptations that beset students in great cities like Chicago, New York, or Philadelphia.

The St. Louis Medical College has not been burned up, or down, as the newspaper press has it.

In those states where legislation for the protection of the profession has recently been indulged, we notice abundant crops of Eclectic, Homeopathic and Female Colleges are being cultivated. The "outsiders" are also forming societies which, under these statutes, have the power of granting certificates which entitle the holders to practice medicine and surgery. We hope some professional wiseacre will find a practical remedy for these little *practical* difficulties in medical legislation.

It is said that bodies injected with a mixture of three parts of glycerine and one of carbolic acid will be preserved several months without giving off any offensive odor.

The first number of the *Journal of the Gynæcological Society of Boston* has come to hand, and well sustains the promise of its prospectus. Published by James Campbell, 18 Tremont street, Boston. $8.00 a year in advance.

The venerable and celebrated Charles D. Meigs, M.D., for many years professor of obstetrics in Jefferson Medical College, and widely known for several standard works in his department, died suddenly at his country seat in Delaware county, Pa., June 22d.

An exchange mentions as a substitute for the stethoscope, in emergency, a glass lamp chimney. Wouldn't a good pair of ears answer better?

Dr. Peters prefers *Spirits Nitri Dulcis* to Belladonna as prophylactic of Scarlatina. He says belladonna is unreliable in small doses, and dangerous in large quantities. To which he might have added, that it probably has no relation whatever to the disease.

The *Philadelphia Reporter* says that saw-dust pills are excellent for many patients, provided they make their own saw-dust.

Asiatic cholera is said to have appeared among the troops in Cuba. Whether this is true or not, we fervently trust both public and private authorities may "put their houses in order," and not neglect their out-houses, barns, streets and alleys.

Dr. Frankland states (*Med. Gazette*) that soft water passing through animal charcoal drives from the latter sufficient phosphate of lime to destroy its property of acting upon lead.

That pretty physiological toy, the sphygmograph, has been modified by Dr. Hawksley, so as to indiate the dynamics both of the respiration and the circulation. He calls his instrument the *stetho-sphygmograph.* The *utero-sphygmograph* is next proposed, with a possible means of diagnosis not only of the activity but the sex of the embryo. Several crowned heads in the old world are anxiously awaiting the results of the experiments about to be instituted.

A writer in the *Sunday Times* of this city has revived the stale claim of Chas. T. Jackson, of Boston, as the originator of anæsthesia by ether for surgical purposes. The late very alphabetical Dr. Morton, notwithstanding his personal qualities however objectionable (*de mortuis nil, etc.,*) must, during all time to come, receive this great honor. Jackson had little if anything more to do with it than the hundreds of medical students and others who had inhaled the ether "for fun" years before its practical application by Morton. The present writer had a contemporaneous knowledge of all the facts in the case, and knows whereof he affirms. He had seen it given by inhalation as a "diffusible stimulant" during surgical operations times almost without number years before its full anæsthetic effect was illustrated even by Morton. Poor Wells even had more claims in this direction than the notable Jackson, who certainly needs some higher claims to distinction than he acquired by his geologic and metallurgic investigations in the Lake Superior regions.

Dr. Henry M. Field, in the *Gynæcological Journal* strongly commends the arseniate of iron in the constipation attending uterine disease. Oxalate of iron is also commended.

Dr. Gerould (*id.*) recomends *Bromide of Iodine* as an energetic therrpeutic agent. He uses it diluted with six parts of Glycerine, as a local application to the uterus; also with the Atomizer, for bronchial and pulmonary difficulties. Sometimes he combines it with crude (unrefined petroleum) Mecca oil. In a case, reported, of enlarged cervix, elongated and os patulous, the fundus anteflexed, its walls thickened, and very sensitive he used this local application, and constitutionally.

℞. Pyrophosphite of Iron, } ℥*ss*;

Bromide of Potassium, }

Aqua, ℥ *oj. M.*

A teaspoonful three times a day.

A writer in the *Philadelphia Reporter* recommends "Dr. Brinsmade's Diaphoretic Powder" as an improvement upon Tully's modification of Dover's Powder, and indeed claims its eligibility over any other anodyne powder:

℞. Morphiæ Sulph., ʒ*j*;

Camphor,

Cretæ Prep.,

Sacch. Alb., *aa.* ʒ*xx*;

*Misceintim*—Ten grains contain very nearly one-sixth of a grain of the Morphia. Given in a teaspoonful of cold water.

A resolution was entertained by the American Medical Association, that any physician who dispenses his own medicines in a place where there is a druggist, shall be sent into professional Coventry, branded as irregular. Another one, that he who contracts with families to do their medical business by the year, shall be hurled into the limbo of outer professional darkness. It is rumored that a uniform, in which the horse-hair wig, gold-headed cane and spectacles are to be conspicuous, is under consideration; also, the question of equipage: as to two, three, or four-wheeled carriages, and as to single horses or spans, and in either case whether particolors are permissible. The New York physicians, it is said, are seriously exercised on the horse question, aside from colors, as Mr. Bergh has entered his protest against geldings, involving, as they do, violation of the statute in regard to cruelty to animals. Boston physicians are equally solicitous as to the other gender of the quadrupeds, since the publication in the papers by a Lowell physician, proving that obscure Malthusian causes are in operation in New England, tending to keep down, not only the excess, but even the present number of population. We expect both of these serious matters will be referred to appropriate committees at the next meeting of the national body. *Vive la bagatelle!*

The *Philadelphia Reporter* glorifies modestly on reaching its twenty-first volume. We congratulate our valued contemporary on its success and prospects.

Dr. Hayes reports, in the *New Orleans Journal of Medicine*, a case of *persistent priapism* successfully treated by Bromide of Potassium. He says: "The pathology of persistent priapism may, *a priori*, be ascribed to, or consist in an *erethism* or *morbid sensibility* or *irritability* of the *prostatic portion of the urethral canal.* Inflammation, extravasation, effusion of lymph, etc., being, when they occur, merely secondary complications. Under our own observation this symptom has occurred in connection with Bright's Disease, Spinal Meningitis, Cerebral Softening; in one instance an encysted tumor in the brain, very often in badly treated Gonorrhœa, chronic cystitis, rectitis—especially when involving that excess of mucus secretion which permits the presence of entozoa—and in numerous other cases of reflex action. Abundantly, it will be found to resist Bromide of Potassium, and the only hope will be first to ascertain, "*What is the matter?*"

